# Individualized MRI-based stroke PRediction scOre using plaque Vulnerability for symptomatic carotid artEry disease patients (IMPROVE)

**DOI:** 10.21203/rs.3.rs-4918579/v1

**Published:** 2024-08-16

**Authors:** Kelly PH Nies, Luc JM Smits, Sander MJ van Kuijk, Akram A Hosseini, Dianne HK van Dam-Nolen, Robert M Kwee, Yoshitaka Kurosaki, Iris Rupert, Paul J Nederkoorn, Pim A De Jong, Daniel Bos, Sen Yamagata, Dorothee P Auer, Andreas Schindler, Tobias Saam, Robert J van Oostenbrugge, M Eline Kooi

## Abstract

**Objective:** In TIA and stroke patients with carotid stenosis, estimations of future ipsilateral ischemic stroke risk and treatment decisions are currently primarily based on the degree of stenosis. Intraplaque hemorrhage (IPH), which can be readily visualized on carotid MRI, is increasingly established as an easy to assess and a very strong and independent predictor for ipsilateral stroke risk, stronger than any clinical risk factor. We developed a clinical prediction model (IMPROVE) incorporating IPH, degree of stenosis, and clinical risk factors to select patients with symptomatic carotid stenosis at high risk for stroke. **Methods:** IMPROVE was developed on pooled clinical and MRI data from five cohort studies of 760 recent TIA or minor stroke patients with carotid plaque who received optimal medical treatment. We used Cox proportional hazards models to determine the coefficients of IMPROVE. IMPROVE was internally validated using bootstrapping and converted to one- and three-year ipsilateral ischemic stroke risk. **Results:** The development dataset contained 65 ipsilateral incident ischemic strokes that occurred during a median follow-up of 1.2 years (IQR: 0.5-4.1). The IMPROVE model includes five predictors, which are in order of importance: degree of stenosis, presence of IPH on MRI, classification of last event (cerebral vs ocular), sex, and age. Internal validation revealed a good accuracy (C-statistic: 0.82; 95% CI: 0.77–0.87) and no evidence for miscalibration (calibration slope: 0.93). **Interpretation:** Using presence of IPH on MRI and only four conventional parameters, the IMPROVE model provides accurate individual stroke risk estimates, which may facilitate stratification for revascularization.

### Introduction

Selection of symptomatic patients for carotid revascularization relies primarily on the degree of stenosis since patients with severe (70–99%) stenosis were identified to benefit most from this procedure in large clinical trials performed in the 1980s-1990s.[1] Benefit of carotid endartectomy (CEA) was considered moderate for patients with 50–69% carotid stenosis and non-existent for mild (< 50%) stenosis.[1,2] However, the 3-year rate of ipsilateral stroke on modern medical management is still 7.4% for mild stenosis.[3] With up to 17 CEAs needed to prevent one future ipsilateral stroke and a ~ 2.7% procedural stroke and death risk, the selection of patients to undergo CEA should be optimized.[1,4]

Clinical prediction models

There are currently three prediction models for the long-term (> 90 days) prediction of ischemic stroke risk in patients with symptomatic carotid stenosis: the European Carotid Surgery Trial (ECST) medical model and its derivative the Carotid Artery Risk (CAR)-score, the Symptomatic Carotid Atheroma Inflammation Lumen stenosis (SCAIL) score, and the CaroTID-VasC score[2,5,6] Critical appraisal showed that the predictive performance of the ECST model and SCAIL score were at high risk of bias.[5] The models were developed based on either a small events per variable (EPV) far below the generally advised 10 outcomes per candidate predictor (SCAIL and CaroTID-VasC)[7], or using data of large, though relatively, old trials from the1980s-1990s (ECST model). Since then significant improvements in medical management (e.g. statin and anti-platelet therapy) for stroke prevention were introduced, for which the ECST model was recalibrated resulting in the derivative CAR-score.[2,8] While there are no sufficiently sized external validations of any of the models with recent data, there are indications of poor-to-moderate performance with a C-statistic of 0.52–0.67 for ECST/CAR and 0.66 for SCAIL.[9–11] The CaroTID-VasC score was developed based on only 20 recurrent events (12 ipsilateral TIAs, 5 ipsilateral strokes, 3 cardiovascular deaths) in 99 symptomatic patients. While no external validation was performed, the small EPV and the majority of outcomes being TIAs and cardiovascular deaths, will make the CaroTID-VasC score less likely to predict ipsilateral ischemic strokes. Therefore, there is a clear need for a more valid tool for risk stratification of patients with symptomatic carotid stenosis.

Rationale for developing the IMPROVE prediction model

Intraplaque hemorrhage (IPH), a key contributor to plaque vulnerability, is present in ~ 50% of carotid plaques in patients with symptomatic carotid stenosis.[12] Carotid plaque MRI is a novel diagnostic tool that enables the identification of IPH[13], which can be recognized as a hyper-intense signal in the bulk of the plaque on a dedicated hyper T1-weighted MR images.[14] IPH is a strong independent predictor of ischemic stroke with hazard ratios (HR) between 8.1 (95% CI: 3.7–17.9) and 10.2 (95% CI: 4.6–22.4), stronger than all conventional clinical risk factors including degree of stenosis.[12,15]

We developed the 'Individualized MRI-based stroke PRediction scOre using plaque Vulnerability for patients with symptomatic carotid artEry disease (IMPROVE)'. This risk score includes IPH presence on carotid plaque MRI and traditional predictors, to enable improved risk stratification for personalized clinical decision-making in patients with symptomatic carotid stenosis.

## Methods

We followed the Transparent Reporting of a multivariable prediction model for Individual Prognosis Or Diagnosis (TRIPOD) guidelines (Supplemental Table).[16]

Data and participants

Studies eligible to contribute to the development dataset were identified based on previously reported search criteria[12], updated to include publications until January 2022. All identified studies received an invitation to participate. Individual patient data from five cohorts, which performed carotid plaque MRI in symptomatic patients with carotid stenosis on optimal medical treatment (OMT) regimen, were pooled. Details of the included studies are presented in the Supplemental Methods. OMT entailed lifestyle advice and medication including statin, antiplatelet, and blood pressure control. The inclusion period ranged from 2002–2015 with follow-up until 2020. Index events included amaurosis fugax, TIA, or minor stroke < 6 months prior to inclusion. Patients had a carotid plaque of ≥ 2mm and up to 99% stenosis (North American Symptomatic Carotid Endarterectomy (NASCET) criteria).

This study utilized data from four previously published cohort studies included in the meta-analysis by Schindler et al., as well as data from the PARISK cohort.[9,12] Ethics Approval Statements: PARISK: Institutional review board approval was obtained from the Medisch Ethische Commissie azM/UM (approval number NL29116.068.09/MEC 09-2-082), and all patients provided written informed consent.[9] Schindler et al.: Corresponding, first, and last authors of publications from eligible cohorts were invited to share anonymized individual patient-level data. No imaging raw data were collected. The local ethics committee waived the requirement for individual consent or specific approval for this analysis.[12]

Outcome

The IMPROVE-predicted outcome is the one- and three-year risk of ipsilateral ischemic stroke, defined as a rapidly developing syndrome of focal cerebral dysfunction lasting > 24h or leading to earlier death, with no other apparent cause than cerebral ischemia. Researchers of all studies who conducted the follow-up patient interviews, were blinded from imaging data. Clinical events were verified by a local stroke or neurology physician and confirmed as ischemic by computed tomography or MRI.

Predictors

A preselection of candidate predictors was performed based on previously reported hazard ratios and/or odds ratios.[2,12,15] Candidate predictors included age, sex, degree of carotid stenosis, time since the last event, classification of the last event (ocular vs cerebral), presence of IPH on MRI, diabetes, hypertension, and hypercholesterolemia. A panel of international experts (n = 17) was consulted on predictor prioritization and availability to ensure face validity.

The time since the last event was defined as the number of days between the index event and carotid plaque MRI. The classification of the index event was categorized into cerebral (stroke/TIA) and ocular (amaurosis fugax).

The category of degree of stenosis (< 50%, 50–69%, 70–99%, based on NASCET criteria) was determined using computed tomography angiography, contrast-enhanced magnetic resonance angiography, or Doppler ultrasonography. On carotid plaque MRI, IPH presence was scored by local observers blinded to clinical data and patient outcome using $T_1$-weighted 3D gradient echo images that were acquired on ≥ 1.5T scanners in all cohorts. IPH was defined as a hyperintense region in the plaque compared to surrounding muscle tissue. The plaque was considered IPH-positive when the signal intensity (SI) ratio was > 1.5[17,18] or > 1.2[19], while remaining studies did not enforce a minimum SI threshold.[9,20]

## Statistical analysis

Baseline patient characteristics were summarized using descriptive statistics after testing for normality using QQ-plots. Normally distributed values are presented as mean (± standard deviation), while otherwise values are presented as median (interquartile range). Missing predictor values were imputed using multiple imputations by chained equations (MICE).

Given the general rule-of-thumb, we allowed for a maximum of 1 predictor (including extra degrees of freedom) per 10 events.[7] All selected predictors were entered into a Cox proportional hazards model with time-to-ipsilateral ischemic stroke as an outcome. The final model was pooled from the multiple imputed datasets according to Rubin's rules. The proportional hazards assumption was tested with global and individual tests on the Schoenfeld residuals.

Internal validation and calibration was performed through bootstrapping with 200 samples. We corrected the regression coefficients for overfitting and estimated optimism of model performance. Individual risk prediction was converted to one and three-year risk of ipsilateral stroke by means of the linear predictor using mean-centered predictor values and the cumulative baseline survival. Conditional survival was estimated using the time since the last event. Model performance was assessed by measures of discrimination and calibration using the concordance (C)-statistic and the calibration curve. The C-statistic is an indication of discriminative performance of the model, for which a value of 0.5 indicates a model that functions no better than a coin toss, while a C-statistic of 1 implies a perfect model. Details on model development and validation are provided in the Supplemental Methods.

## Risk groups

Patients were categorized into high or intermediate risk based on the rounded median ipsilateral ischemic stroke risk according to the IMPROVE model. Patients were considered to be at high risk when exceeding the median 3-year risk of ipsilateral ischemic stroke, while remaining patients were categorized at intermediate risk. Risk groups are presented in Kaplan-Meier plots accompanied by the log-rank test. The sensitivity, specificity, positive predictive value (PPV) and negative predictive value (NPV) of IMPROVE was determined. The performance was compared to a care-as-usual scenario where all patients with ≥ 50% carotid stenosis were considered at high risk as recommended in guidelines from the European Society for Vascular Surgery.[21]

## Results

Participants

Data from 794 symptomatic patients with a carotid plaque of ≥ 2 mm thickness were pooled to form the development dataset (Fig. 1). Thirteen patients were excluded based on the index event (6 contralateral strokes or TIAs, 2 posterior circulation strokes) or occlusion of the ipsilateral carotid artery (n = 5). Patients (n = 2) who experienced simultaneous bilateral strokes during follow-up were excluded to prevent bias from potential cardioembolic strokes. Given that IPH presence was a crucial predictor, patients with missing IPH status were excluded (n = 17) in addition to patients with missing follow-up (n = 2). This resulted in a development dataset with in total 760 patients who had a total follow-up of 1825 years and a median follow-up of 13.8 (IQR: 6.0-48.6) months.

The pooled dataset contained 760 patients of whom 68% were males (Table 1). The degree of carotid stenosis was < 50%, 50–69%, or ≥ 70% in 48%, 27%, and 25% of the patients, respectively. Seventy percent of the patients were hypertensive and 54% used statins before the index event. One hundred forty-one patients experienced a total of 142 ipsilateral ischemic cerebrovascular events during a median follow-up duration of 13.8 (IQR: 6.0-48.6) months, of which 65 were strokes, 57 TIAs, and 20 amaurosis fugax (Table 2). Considering 65 primary outcomes (ipsilateral ischemic strokes), a maximum of six predictors could be selected for model development to prevent overfitting.[7]

Table 1
Patient characteristics of the pooled development dataset

| Characteristic | Overall N = 760 | Hosseini 2013 N = 179 | Hosseini 2017 N = 149 | Kurosaki 2011 N = 82 | Kwee 2012 N = 126 | Van Dam-Nolen 2022 N = 224 |
|---|---|---|---|---|---|---|
| Age | 72 (± 10) | 72 (± 10) | 75 (± 10) | 77 (± 8) | 69 (± 10) | 69 (± 9) |
| Male sex | 520 (68%) | 127 (71%) | 88 (59%) | 70 (85%) | 79 (63%) | 156 (70%) |
| Index event | | | | | | |
| • TIA | 298 (39%) | 78 (44%) | 58 (39%) | 16 (20%) | 47 (37%) | 99 (44%) |
| • Stroke | 364 (48%) | 63 (35%) | 73 (49%) | 65 (79%) | 63 (50%) | 100 (45%) |
| • Amaurosis Fugax | 98 (13%) | 38 (21%) | 18 (12%) | 1 (1.2%) | 16 (13%) | 25 (11%) |
| Time since last event (in days) | 40 (± 35) | 56 (± 47) | 35 (± 34) | 1 (± 5) | 33 (± 18) | 50 (± 23) |
| Degree of stenosis | | | | | | |
| • 0–49% stenosis | 344 (48%) | 0 (0%) | 62 (42%) | 22 (27%) | 82 (65%) | 178 (95%) |
| • 50–69% stenosis | 198 (27%) | 68 (38%) | 71 (48%) | 6 (7.3%) | 44 (35%) | 9 (4.8%) |
| • 70–99% stenosis | 181 (25%) | 111 (62%) | 16 (11%) | 54 (66%) | 0 (0%) | 0 (0%) |
| IPH presence on MRI | 351 (46%) | 114 (64%) | 54 (36%) | 59 (72%) | 37 (29%) | 87 (39%) |
| Current smoker | 231 (30%) | 106 (59%) | 36 (24%) | 10 (12%) | 29 (23%) | 50 (22%) |
| Hypertension | 515 (70%) | 142 (79%) | 121 (81%) | 60 (73%) | 27 (21%) | 165 (83%) |
| Diabetes Mellitus | 169 (23%) | 20 (11%) | 33 (22%) | 35 (43%) | 31 (25%) | 50 (25%) |
| History of ischemic CVD | 156 (33%) | unknown | unknown | 35 (47%) | 31 (25%) | 47 (21%) |
| Statin use before index event | 462 (61%) | 140 (78%) | 76 (51%) | 21 (26%) | unknown | 109 (49%) |

| Characteristic | Overall N = 760 | Hosseini 2013 N = 179 | Hosseini 2017 N = 149 | Kurosaki 2011 N = 82 | Kwee 2012 N = 126 | Van Dam-Nolen 2022 N = 224 |
|---|---|---|---|---|---|---|
| Values are presented as n (%) or mean (± standard deviation) given that all continuous variables were normally distributed. Missing values include history of ischemic cardiovascular disease (n = 293) degree of stenosis (n = 34), hypertension (n = 20), diabetes mellitus (n = 16), time between MRI and index event (n = 5), and statin use before index event (n = 1). CVD = cardiovascular disease, IPH = intraplaque hemorrhage. | | | | | | |

Table 2
Ipsilateral ischemic cerebrovascular event recurrence during follow-up

| | Overall N = 760 | Hosseini 2013 N = 179 | Hosseini 2017 N = 149 | Kurosaki 2011 N = 82 | Kwee 2012 N = 126 | Van Dam-Nolen 2022 N = 224 |
|---|---|---|---|---|---|---|
| Follow-up (in months) | 13.8 (6.0–48.6) | 2.2 (0.8–15.2) | 20.2 (11.3–32.0) | 9.5 (2.0–18.8) | 12* | 61.7 (38.5–69.4) |
| Ipsilateral ischemic cerebrovascular events | 141 (19%) | 62 (35%) | 20 (13%) | 11 (13%) | 13 (10%) | 35 (16%) |
| ● Stroke | 65 (8.6%) | 26 (15%) | 14 (9.4%) | 7 (8.5%) | 3 (2.4%) | 15 (6.7%) |
| ● TIA | 57 (7.5%) | 25 (14%) | 3 (2.0%) | 4 (4.9%) | 9 (7.1%) | 16 (7.1%) |
| ● Amaurosis Fugax | 20 (2.6%) | 11 (6.1%) | 3 (2.0%) | 0 (0%) | 1 (0.8%) | 5 (2.2%) |

Values are presented as n (%) or median (IQR) given that all continuous variables were non-normally distributed. The first ipsilateral ischemic cerebrovascular event to occur during follow-up are presented. *All patients had a one-year follow-up. TIA = transient ischemic attack.

Predictor selection

The following predictors were selected based on previously reported hazard ratios and expert opinion: age, sex, classification of the last event (ocular vs cerebral), degree of carotid stenosis, and the presence of IPH on MRI (Supplemental Results). Time since the last event was not included as a predictor in the IMPROVE model, however the variable was used to determine the conditional stroke probability given the stroke-free time between the last event and risk calculation.

Information on all predictors was complete for 678 patients (Supplemental Results). Only for degree of stenosis there were some absent values (n = 34). The predictors showed no strong correlations with each other and the proportional hazards assumption was not rejected (Supplemental Results). Consequently, all selected predictors were included in the model. Data of all 760 patients was used for model development after multiple imputation of missing values.

The IMPROVE model

The hazard ratios of the IMPROVE predictors are presented in Fig. 2. IPH presence had an HR of 5.61 (95% CI: 2.92–10.77). The degree of stenosis had a HR of 4.54 (95% CI: 2.46–8.38) and 7.42 (95% CI: 3.45–15.95) for 50–69% and 70–99%, respectively. The classification of the index event as cerebral versus ocular had a HR of 3.72 (95% CI: 1.11–12.52). Male sex contributed with a HR of 1.26 (95% CI: 0.64–2.48). Age contributed with a HR of 1.14 (95% CI: 0.84–1.55) per 10-year increase.

Model performance

The IMPROVE score exhibited good discriminative value in the development dataset with a C-statistic of 0.84 (95% CI: 0.78–0.88). After internal validation, model performance decreased only marginally to 0.82.

Overall, calibration of the model showed good alignment between observed and predicted ipsilateral ischemic stroke risk with a calibration slope of 0.93 (Supplemental Results). Time-dependent calibration showed a calibration slope of 0.93 and 0.94 for 1 and 3-year ipsilateral ischemic stroke-free survival, respectively (Fig. 3).

## Estimating the risk of ipsilateral ischemic stroke using IMPROVE

To determine ipsilateral ischemic stroke risk in percentages for clinical use, cumulative baseline survival and formulae for survival predictions are provided (Supplemental Results).

Patients had a mean 1 and 3-year ipsilateral ischemic stroke risk of 2.13% and 8.16%, respectively. Several examples of individual IMPROVE risk estimations are presented in Table 3. A clear example of a high-risk individual is a 73-year old male patient with a recent cerebral stroke and an IPH-positive carotid plaque causing ≥ 70% stenosis, resulting in an IMPROVE-estimated ipsilateral ischemic stroke risk of 16.5% within one year and 51.6% within 3 years. An individual considered for carotid revascularization according to current guidelines, i.e. a 73-year old male with ≥ 70% stenosis, an ocular index event, and no IPH, has an estimated 3-year risk of only 4.2%. On the other hand, patients with < 50% carotid stenosis at relatively high risk of stroke recurrence can be identified. A 73-year old male patient with a cerebral index event who is IPH-positive has a 3-year risk of 10.6%. For an 80-year old patient with these characteristics, this risk would be 11.4%.

Table 3
Examples of IMPROVE-based ipsilateral ischemic stroke risk

| Carotid stenosis | Sex | IPH | Index event | IMPROVE 1-year risk (%) | IMPROVE 3-year risk (%) |
|---|---|---|---|---|---|
| > 70% | male | Yes | cerebral | 16.5 | 51.6 |
| | | No | cerebral | 3.6 | 13.6 |
| | | No | ocular | 1.1 | 4.2 |
| | female | Yes | cerebral | 13.5 | 44.2 |
| | | No | cerebral | 2.9 | 11.1 |
| | | No | ocular | 0.9 | 3.4 |
| 50–69% | male | Yes | cerebral | 10.8 | 36.8 |
| | | No | cerebral | 2.3 | 8.8 |
| | | No | ocular | 0.7 | 2.7 |
| | female | Yes | cerebral | 8.7 | 30.9 |
| | | No | cerebral | 1.8 | 7.2 |
| | | No | ocular | 0.5 | 2.2 |
| < 50% | male | Yes | cerebral | 2.8 | 10.6 |
| | | No | cerebral | 0.6 | 2.2 |
| | | No | ocular | 0.2 | 0.7 |
| | female | Yes | cerebral | 2.2 | 8.7 |
| | | No | cerebral | 0.5 | 1.8 |
| | | No | ocular | 0.1 | 0.5 |

Overview of ipsilateral ischemic stroke risk for exemplary patients of 73-years old.

## IMPROVE risk groups

The median IMPROVE-estimated 3-year ipsilateral stroke risk was 9.4% (IQR: 2.0-20.7%) in the development dataset. Thus, patients with an ipsilateral stroke risk of ≥ 9% were categorized into a high-risk profile, while remaining patients were considered to be at intermediate risk. These risk groups showed a clear division in ipsilateral stroke-free survival (Fig. 4). When stratifying patients using a care-as-usual scenario where all patients ≥ 50% were considered as high risk, 5.3% of intermediate risk patients with < 50% stenosis had a recurrent ipsilateral ischemic stroke within 3 years. In comparison, IMPROVE stratification of patients resulted in a 3-year ipsilateral ischemic stroke recurrence on OMT for only 2.1% of the patients with an intermediate risk profile. IMPROVE also increased the likelihood of identifying individuals at true high risk of ipsilateral stroke recurrence compared to care-as-usual. Patients identified as high risk by IMPROVE had a larger percentage of ipsilateral ischemic strokes within 3 years (24.0% vs. 20.7%, respectively).

The sensitivity of IMPROVE at the 9% threshold for ipsilateral ischemic stroke recurrence within 3 years was 92.6% (95% CI: 90.7–94.5). In comparison, care-as-usual risk stratification based on ≥ 50% carotid stenosis, resulted in a sensitivity of 80.6% (95% CI: 77.8–83.4). The specificity of IMPROVE at the 9% threshold was 54.2% (95% CI: 50.7–57.8) versus 52.9% (95% CI: 49.3–56.4) for care-as-usual.

To investigate model stability, several alternative models with varying predictor selection methods, subpopulations of the development dataset, and outcomes of interest were explored and all showed similar performance to the IMPROVE model (Supplemental Results).

## Discussion

We developed a novel clinical prediction tool (IMPROVE). IMPROVE is the first sufficiently powered clinical prediction model of ipsilateral ischemic stroke risk for patients with symptomatic carotid artery disease on current optimal medical treatment. IMPROVE exhibited good performance after internal validation. It demonstrates the importance to look beyond the degree of stenosis for risk stratification, since the ipsilateral ischemic stroke risk can vary to a large extent between patients with similar degree of stenosis.

For the development of the score, individual patient data from five cohort studies were pooled to generate a derivation cohort with a representative sample of patients with carotid artery stenosis who present with a recent amaurosis fugax, TIA, or minor stroke, comparable to the patient characteristics of previous large clinical trials (i.e. ECST) [1]. However, the median age in our cohort is considerably higher than the previous large trials, due to demographic changes since the 1980s-1990s when the ECST trial was performed.

The performance of IMPROVE in the derivation cohort with a C-statistic of 0.84 (95% CI: 0.78–0.88) was marginally superior to the performance of SCAIL (0.82 (95% CI: 0.66–0.97). While the SCAIL score already demonstrated the potential of using plaque vulnerability for improving risk prediction, external validation of SCAIL resulted in a C-statistic of 0.66 (95% CI: 0.51–0.80).[11] The large decline in discriminative performance of SCAIL may be explained by the very low EPV of < 2, considerably lower than the recommended minimum of 10 EPV that was used to develop IMPROVE.[7] The CaroTID-VasC score had a C-statistic of 0.88 (95% CI: 0.81–0.96) in the derivation cohort, which declined to 0.83 after internal validation.[6] Note that the CaroTID-VasC score was developed in 99 patients with only 25% of included outcomes-of-interest consisting of ipsilateral ischemic strokes. In comparison, IMPROVE was based on ipsilateral ischemic stroke only with 65 events in 760 patients. In addition to the low EPV of 4 for CaroTID-VasC, an univariate analysis and stepwise selection of candidate predictors was used for the development of this score. This can lead to overfitting so that the model is expected to show lower performance in other datasets. The ECST model/CAR score had only moderate discriminative performance (C-statistic: 0.67 [95% CI: 0.54–0.80]) in the Plaque At RISK (PARISK) study of 244 symptomatic patients with < 70% carotid stenosis.[9] Additionally, in a study of 134 patients with severe carotid stenosis no association was found between the ECST model and recurrent cerebrovascular events (HR = 0.86; 95% CI: 0.45–1.65; P = 0.65).[10]

The SCAIL score is based on the degree of stenosis and the maximal standard uptake value of carotid plaque on $^{18}$F-fluorodeoxyglucose Positron Emission Tomography (PET), an indicator of plaque inflammation.[22] The radioactive tracer typically needs to be ordered a few days in advance, making SCAIL practically challenging. In contrast, the presence of IPH for IMPROVE is much easier to obtain with a short 5-minute additional scan with a standard neurovascular coil during a carotid or brain MRI examination. MRI is much cheaper and more readily available compared to PET. The CaroTID-VasC score also includes plaque vulnerability, which is defined as echolucency on ultrasound for this score.[6] Duplex ultrasound is widely available and relatively cheap. However, the intraobserver agreement for scoring plaque echogenicity is known to be poor.[23] A more objective and less observer-dependent measure for plaque echogenicity is the gray-scale median (GSM).[24] The PARISK study investigated the association between the risk for ipsilateral ischemic symptoms for various imaging modalities in TIA and stroke patients with a carotid plaque. No association between the echogenicity (GSM) and the risk for recurrent ipsilateral cerebrovascular events was reported, while on the contrary IPH was an independent risk factor for ipsilateral ischemic events.[9]

Our study further strengthens the importance of using plaque vulnerability imaging parameters to get insight into the individual risk of patients. The strong contribution of IPH presence on MRI in the IMPROVE tool confirms the importance of incorporating MRI-based carotid plaque features for individualized ipsilateral ischemic stroke risk estimation. While external validation of the IMPROVE model in an independent cohort is of high priority, the IMPROVE score can already be used in clinical practice in border-line cases to provide additional information to patients about their risk and to weigh in this risk on the choice of treatment. The exemplary IMPROVE-threshold of 9% has a high sensitivity of > 90% and a specificity that is also higher than care–as-usual stratification of high-risk patients based on a degree of stenosis ≥ 50%. The optimal risk threshold will depend on the user's goal, such as stratification for carotid revascularization or aggressive novel medication.

Due to the increased sensitivity and specificity, we expect that the IMPROVE model will be superior to care-as-usual for selecting patients for carotid revascularization that will benefit most. Decision-analytic studies are warranted to determine the optimal thresholds of IMPROVE-based personalized clinical decision-making. A randomized trial including a cost-effectiveness analysis is urgently needed to demonstrate whether IMPROVE-based risk stratification for carotid revascularization or novel aggressive medication can lead to a reduction in stroke and/or less costs.

The IMPROVE model has important strengths. Foremost, IMPROVE is the first sufficiently powered model to predict ipsilateral ischemic stroke risk on current OMT incorporating information on clinical risk factors and carotid plaque composition. In contrast, the most common risk score, the ECST model, and its derivative, the CAR score, is based on robust, but outdated data. Second, contrary to the SCAIL and CaroTID-VasC models, IMPROVE complies to the recommended minimum events per variable (EPV) of 10.[25]. Considering the 65 ipsilateral ischemic recurrent strokes in our derivation cohort, the IMPROVE model with 5 predictors and 1 extra degree of freedom due to categorization, has an EPV of [illegible]11. Third, for the development of IMPROVE, we used ipsilateral ischemic stroke only and not TIA and amaurosis fugax as a clinical endpoint. Fourth, information of predictor values in the derivation cohort was near complete. Only the degree of stenosis was missing and this occurred in < 5% of the patients, which was comfortably under the threshold of 10% above which bias is believed to be introduced.[26] Multiple imputation by the MICE procedure was performed, since it can reduce bias by up to 98% compared to complete case analysis even for only 5% of missing values.[27] Fifth, we used preselected predictors based on reported predictor strength in literature and expert opinion. No stepwise selection procedures were used, since they may lead to inflated coefficients, the selection of nuisance variables, and/or exclusion of true variables that are not statistically significant but do have predictive value.[28] The use of preselected predictors not only positively impacts external validity, IMPROVE is thereby also expected to have good face validity since expert opinion on predictor importance was consulted. Last, while the ECST and CaroTID-VasC models were developed only for patients with ≥ 50% stenosis, outward remodeling of the carotid artery frequently occurs and it is also considered as a feature of plaque vulnerability. Therefore, vulnerable plaques can also be present in patients with < 50% stenosis. IMPROVE is the only model that facilitates ipsilateral ischemic stroke prediction for all patients with a carotid plaque ≥ 2 mm. Indeed, we showed that the IMPROVE model can stratify a subgroup of patients with < 50% stenosis that still have a considerable (> 9% 3-year) ipsilateral ischemic stroke risk.

Presently, we present the internal validation of the IMPROVE score. While external validation in an independent sample should be a goal for future research before large-scale implementation of the risk score, the performance of the model is expected to be highly stable. The derivation cohort was based on five cohort studies with patients from varying countries and ethnic backgrounds. Also in the five cohorts, different MRI sequences, different field strengths and MRI systems from different vendors were used and the presence of IPH was scored by local observers. Therefore, the cohort already exhibits great variability and thus the IMPROVE score has been developed on the building blocks of flexibility. We have assessed a number of alternative models developed with backwards selection and in a subset of the development dataset, which showed similar performance to the IMPROVE model, indicating a strong stability of an MRI-based ipsilateral ischemic stroke risk model. Internal validation of the IMPROVE model by means of bootstrapping has shown that the performance of the model decreased only marginally (C-statistic decreased from 0.84 to 0.82), further strengthening our expectation that the model performance will remain of high quality.

The IMPROVE model, utilizing information from novel carotid plaque MRI techniques and clinical risk factors, shows good performance for estimating ipsilateral ischemic stroke risk in symptomatic patients with carotid artery disease. IMPROVE can aid in risk stratification for secondary stroke prevention strategies. We have demonstrated that IMPROVE is able to identify subpopulations of high-risk patients who are conventionally considered at intermediate risk in care as usual, and similarly, intermediate-risk patients with 70–99% stenosis can be identified. External validation and clinical impact analysis are urgently needed towards clinical implementation of IMPROVE.

## Abbreviations

CEA
carotid endarterectomy
HR
hazard ratio
IPH
intraplaque hemorrhage
MRI
magnetic resonance imaging
NNT
number needed to treat
TIA
transient ischemic attack

## Declarations

Author disclosures: The authors declare no competing interests or relationships with industry regarding this manuscript.

Funding disclosures: Prof. M.Eline Kooi is supported by an Aspasia Grant 2018/SGw/00460457 from NWO. Dr. Akram A. Hosseini has received funding from the MRC, UK (grant MR/T005580/1); National Institute of Health/NIA, USA (grant 1R56AG074467-01) through her institute, and honoraria from Biogen, Lilly, and Eisai for teaching and advice on Alzheimer's diagnostics and/or drugs.

## Author contributions

K.P.H.N., L.J.M.S., S.M.J.K., P.J.N., A.S., T.S, R.J.O., and M.E.K. contributed to the conception and design of the study; K.P.H.N., A.A.H., D.H.K.D.N., R.M.K., Y.K., I.R., P.J.N., P.A.J., D.B., S.Y., D.P.A., R.J.O., and M.E.K. contributed to the acquisition and analysis of data; K.P.H.N. contributed to drafting the text or preparing the figures.

## Statement Confirming Availability or Absence of Shared Data Required

The data used in this research were collected subject to the informed consent of the participants. Access to the data can only be granted in line with that consent, subject to approval by the project ethics board and under a formal Data Sharing Agreement.

## Figures

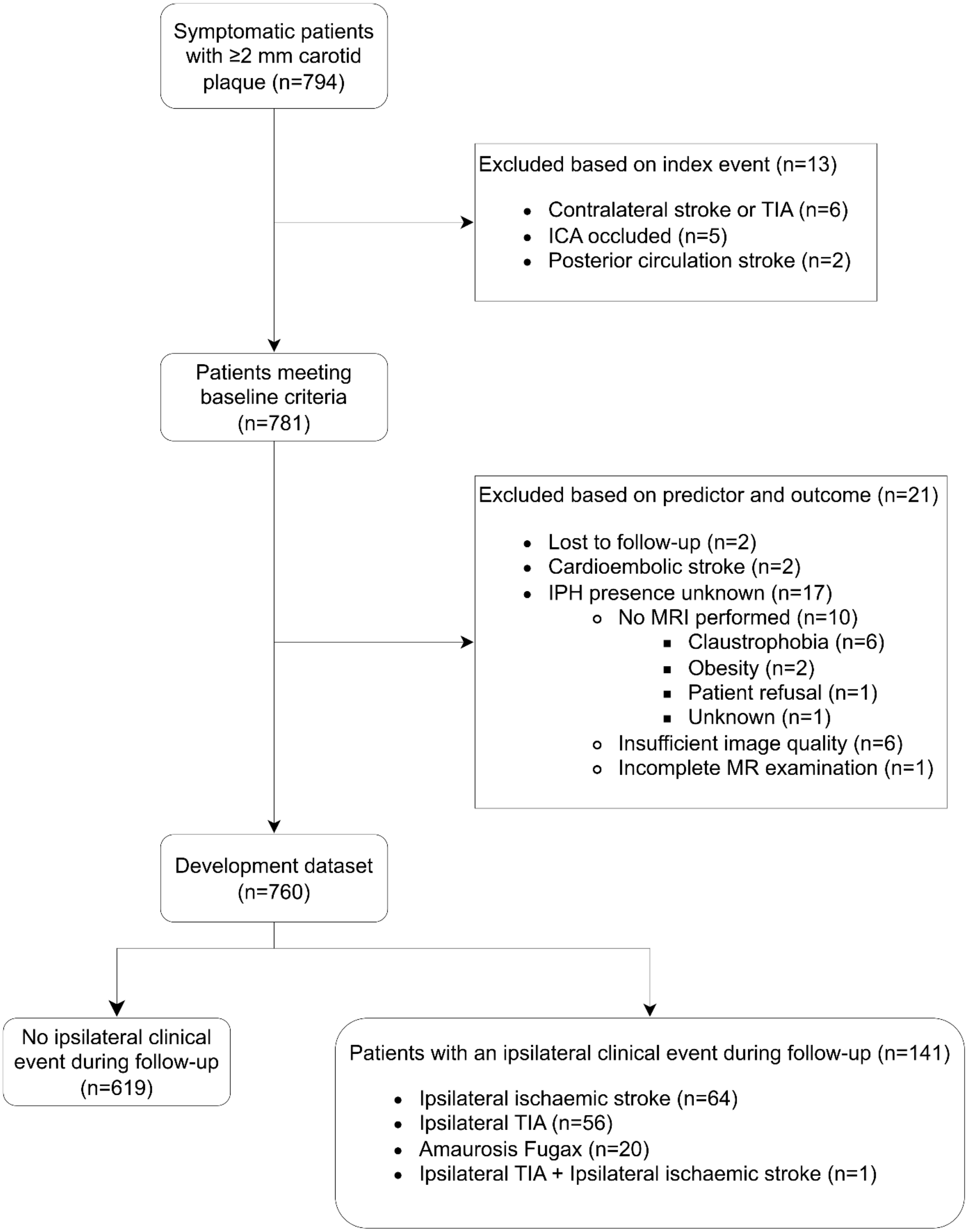

### Figure 1

**Study flowchart**. The selection process of patients from pooled data from 5 cohort studies for the development dataset of the IMPROVE model.

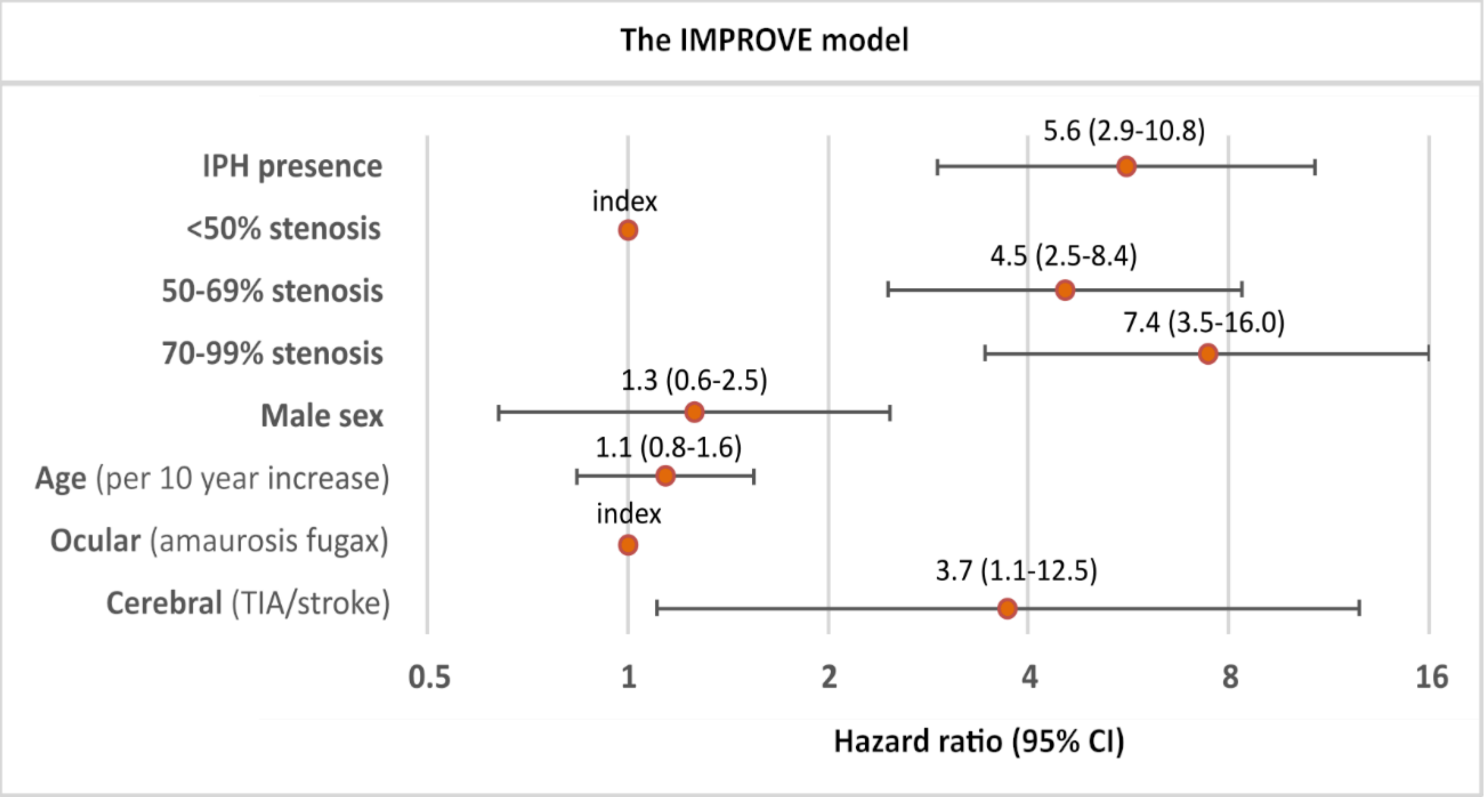

Figure 2

**Specification of the IMPROVE model.**Predictors included intraplaque hemorrhage (IPH) presence on MRI, degree of stenosis, male sex, age, and classification of the last event (ocular vs. cerebral).

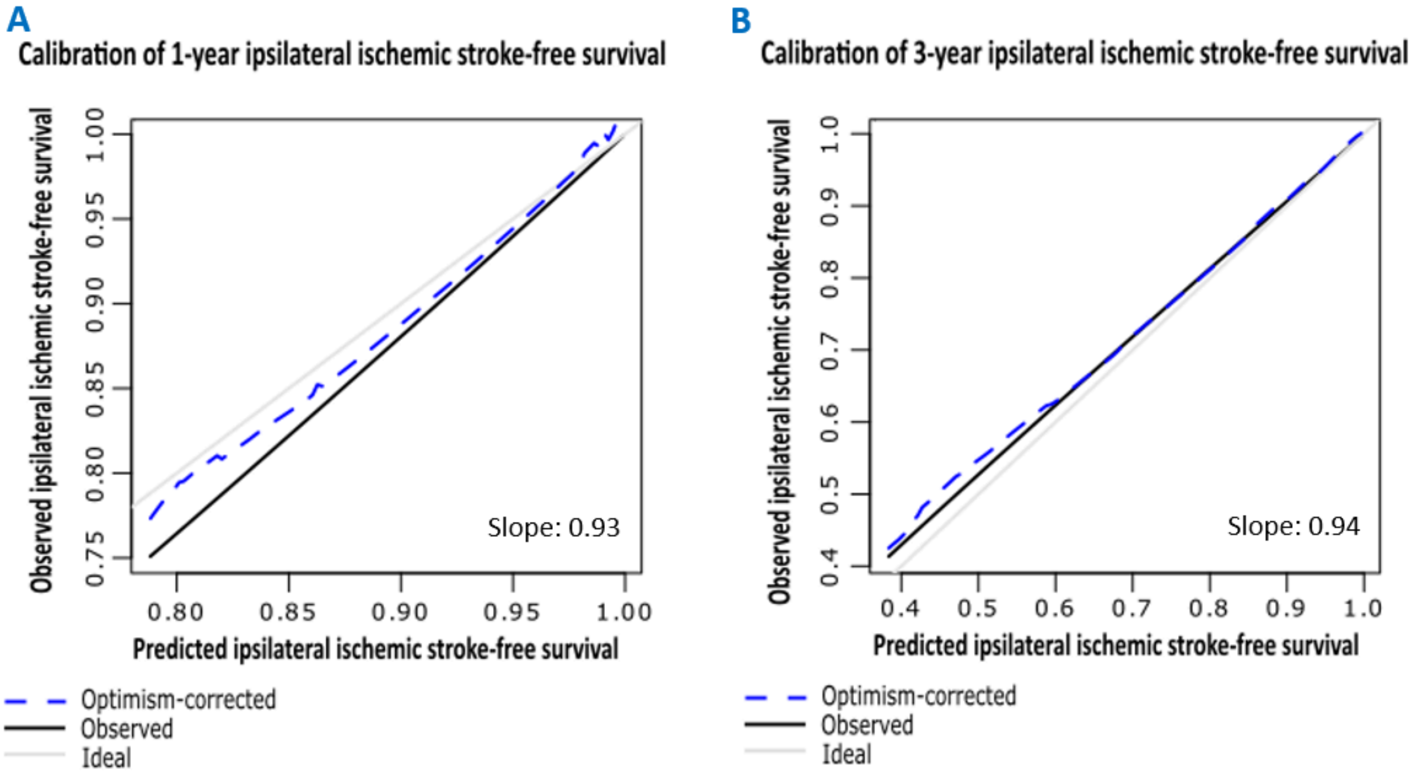

Figure 3

**Calibration curves of the observed versus IMPROVE-predicted risks**. 1 year (**A**) and 3 year (**B**) risk of ipsilateral ischemic stroke illustrate a good calibration of IMPROVE with calibration slopes ≥0.93.

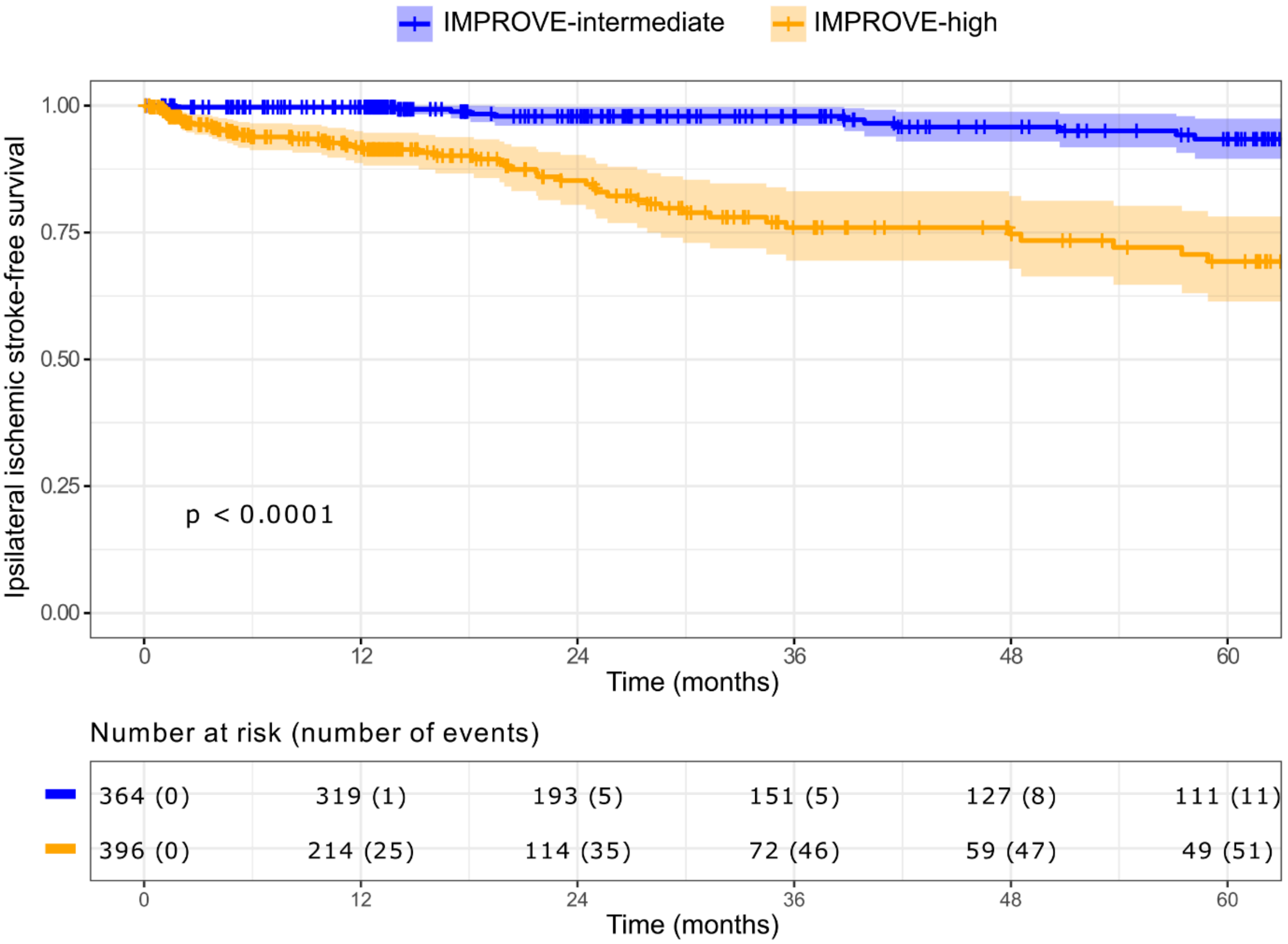

**Figure 4**

**Ipsilateral ischemic stroke-free survival of IMPROVE risk categories**. Patients were stratified using a threshold of ≥9% (high-risk) versus <9% (intermediate-risk) 3-year IMPROVE-based ipsilateral stroke risk. It can be clearly observed that these two categories demonstrate a significant difference in cumulative ipsilateral ischemic stroke-free survival ($p<0.0001$).
